# Supplementary material for: The speed limit of optoelectronics
Source: Nat Commun. 2022 Mar 25;13:1620. doi: 10.1038/s41467-022-29252-1 (PMC8956609; doi:10.1038/s41467-022-29252-1)
Supplement: Supplementary file 1 — Supplementary Information [file 41467_2022_29252_MOESM1_ESM.pdf]

Supplementary Information
for
The speed limit of optoelectronics

M. Ossiander[1, †, *], K. Golyari[1,2], K. Scharl[1,2], L. Lehnert[1,2], F. Siegrist[1,2], J. P. Bürger[1,2], D. Zimin[1,2], J.A. Gessner[1,2], M. Weidman[1,2], I. Floss[3], V. Smejkal[3], S. Donsa[3], C. Lemell[3], F. Libisch[3], N. Karpowicz[4], J. Burgdörfer[3], F. Krausz[1,2, *], M. Schultze[2,5]

1 Max-Planck-Institut für Quantenoptik, Hans-Kopfermann-Str. 1, 85748 Garching, Germany, EU
2 Fakultät für Physik, Ludwig-Maximilians-Universität München, Am Coulombwall 1, 85748 Garching, Germany, EU
3 Institute for Theoretical Physics, Vienna University of Technology, Wiedner Hauptstrasse 8-10, 1040 Vienna, Austria, EU
4 CNR NANOTEC Institute of Nanotechnology, via Monteroni, 73100 Lecce, Italy, EU
5 Institute of Experimental Physics, Graz University of Technology, Petersgasse 16, 8010 Graz, Austria, EU
[†] Current Address: John A. Paulson School of Engineering and Applied Sciences, Harvard University, 29 Oxford St, Cambridge, MA 02138, United States
*Corresponding Authors: mossiander@g.harvard.edu, ferenc.krausz@mpq.mpg.de

Supplementary Figures

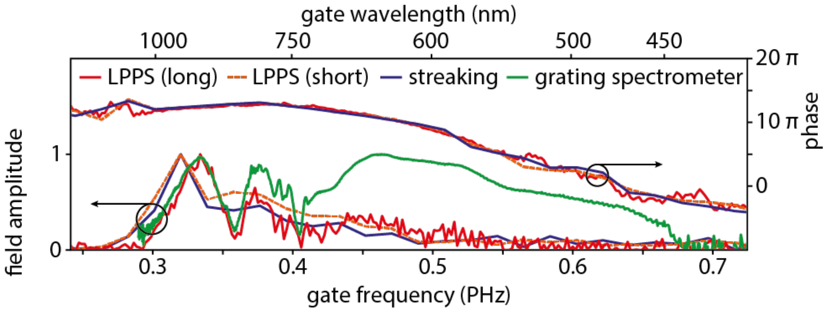

Supplementary Fig. 1.

Spectral-domain retrieval of gate fields.

Spectral amplitude and phase of the electric field of the gate laser pulses retrieved via differentiation of the vector potential retrieved from a linear petahertz photoconductive sampling (LPPS) scan with long and short delay range (red: source-gate-delay-range $-250\text{ fs} \leq \tau \leq 250\text{ fs}$, orange: source-gate-delay-range $-30\text{ fs} \leq \tau \leq 30\text{ fs}$). Spectral amplitude and phase retrieved from attosecond streaking (blue: source-gate-delay-range $-30\text{ fs} \leq \tau \leq 30\text{ fs}$) and the gate field spectral amplitude measured with a calibrated grating spectrometer (green) for comparison.

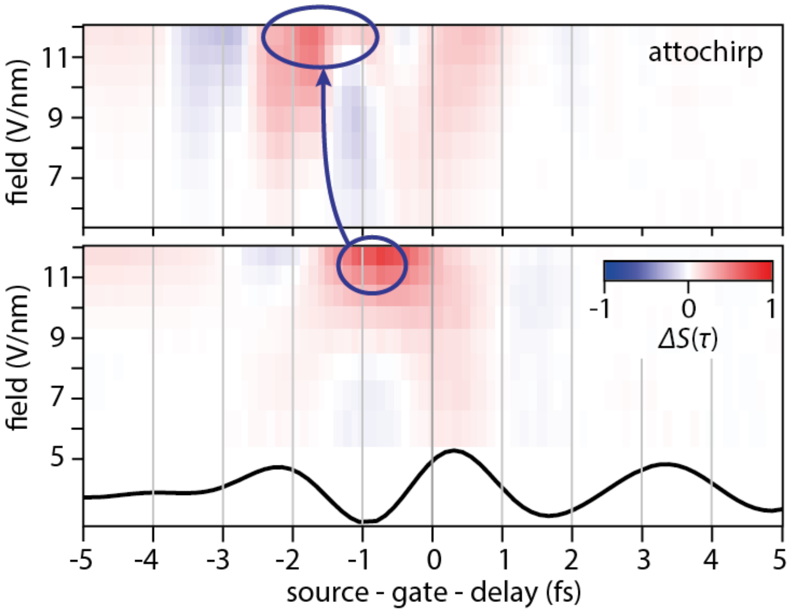

Supplementary Fig. 2.

Effect of VUV-source pulse chirp on the recorded multi-band deviations.

Lower panel: simulated LPPS signal deviation $\Delta S(\tau)$ as a function of source-gate-delay and gate intensity for the experimental gate pulse vector potential (black) when carriers are injected by a compressed VUV source pulse (see Fig. 4b). Top panel: injection by a positively chirped (0.27 fs^2) source pulse shifts the interband transition to earlier time and thus also shifts the timing of the largest $\Delta S(\tau)$, see blue circles.

Supplementary Tables

Extremum	Time	Reference	Signal / Ref.	SD / Ref.	$\Delta S(\tau)$ / SD
1	-2.0 fs	100%	117%	29%	59%
2	-1.0 fs	100%	46%	17%	318%
3	0.4 fs	100%	69%	16%	194%
4	1.6 fs	100%	97%	23%	13%
5	3.4 fs	100%	90%	19%	53%
6	4.8 fs	100%	86%	19%	74%
7	6.2 fs	100%	109%	23%	39%

Supplementary Table 1.
Evaluation of $\Delta S(\tau)$ at the reference maxima for the highest field amplitude in Fig. 3b.
